# The Phycotoxin Domoic Acid as a Potential Factor for Oxidative Alterations Enhanced by Climate Change

**DOI:** 10.3389/fpls.2020.576971

**Published:** 2020-10-30

**Authors:** Joaquin Cabrera, Paula Mariela González, Susana Puntarulo

**Affiliations:** ^1^Universidad de Buenos Aires, Facultad de Farmacia y Bioquímica, Fisicoquímica, Buenos Aires, Argentina; ^2^Instituto de Bioquímica y Medicina Molecular (IBIMOL), Consejo Nacional de Investigaciones Científicas y Técnicas (CONICET)-Universidad de Buenos Aires, Buenos Aires, Argentina

**Keywords:** domoic acid, harmful marine toxins, oxidative stress, photosynthetic organisms, climate change

## Introduction

The climate change affects water quality and sustainable development; therefore, security of the aquatic communities is altered (World Meteorological Organization, 2020). However, data on water resources are patchy and incomplete. Exceptional global heat, retreating ice, and high sea level records driven by greenhouse gases from human activities were described over the last decade. Average temperatures for the 5-year (2015–2019) and 10-year (2010–2019) periods are almost certain to be the highest on record. The scenario is the same in each point of the planet because the heating induced by human activities is affecting the scale and intensity of extreme meteorological phenomena. Levels of heat-trapping greenhouse gases in the atmosphere have reached another high new record in 2018 [37,000 tons of carbon dioxide (CO_2_)], and threat to ocean life is huge because more than 90% of excess heat caused by global change ends up affecting water and aquatic life. Over the last decades, oceans have become more acid (0.1 pH unit) because the increase in dissolved CO_2_ causes this effect (IPCC Intergovernmental Panel on Climate Change, 2007).

Phytoplankton productivity, specifically that of diatoms with a relatively large cell size, is limited by iron (Fe) availability in the high-nutrient, low-chlorophyll (HNLC) region of the ocean (de Baar et al., 2005). Fe availability will change with the increasing contribution of ferrous to ferric Fe (Millero et al., 2009) and the conditional stability constant of Fe-ligand complex (Shi et al., 2010) in response to the increase in the acidity of seawater. Other human perturbations, such as land use and SO_2_ and NO_x_ emissions, will also alter the Fe distribution and bioavailability in the open oceans (Mahowald et al., 2009). The interactive effects of the ocean acidification and the Fe availability are expected to play crucial roles in the biogeochemical cycling of nutrients in the HNLC regions. The elemental composition of unialgal culture of *Pseudo-nitzschia pseudodelicatissima* changed in response to alterations in both CO_2_ levels and bioavailable dissolved inorganic Fe concentrations (Sugie and Yoshimura, 2013). Sugie et al. (2013) reported that high CO_2_ affects nutrient dynamics in Fe-limited phytoplankton communities.

In marine environments, some microalgae (diatoms, dinophyceae, rhodophyte, dinoflagellates), ciliates, and cyanobacteria species synthetize toxins (Reguera, 2002) that diffuse out of the organisms and reach other aquatic systems directly from the water or through the trophic transfer. Continued acidification of the ocean inhibits the growth of phytoplankton species that have shells of calcium carbonate, which dissolve in acidic conditions, and the growth of organisms without calcium carbonate shells is favored (Moore et al., 2008). During harmful algal blooms (HABs), bloom-forming diatom species tend to be more flexible in the use of different carbon sources, and these abilities may provide a competitive advantage, especially under changing conditions as they occur during a bloom. The ocean acidification during blooms favored organisms that fix CO_2_ such as some diatoms (Hansen, 2002). It has been reported that *Pseudo-nitszchia australis* (Wingert, 2017), *P. pseudodelicatissima* (Sugie and Yoshimura, 2013), and *Pseudo-nitszchia subcurvata* increase their growth rate under these conditions. Even more, *Pseudo-nitzschia multiseries* increase the production of the biotoxin domoic acid (DA) (Trimborn et al., 2008). Degraded water quality from nutrient pollution, physical, biological, and other chemical factors contribute to the growth and persistence of many HABs (Cabrera et al., 2019a). The most severe consequences of HABs include effects on fish, bird, and mammal mortalities, by respiratory or digestive tract problems, memory loss, seizures, lesions, and skin irritation (Sellner et al., 2003). Fluctuating seasonally, temperature, oxygen (O_2_) consumption, availability of food, endogenous rhythms, and HABs are among the important potential stressors studied for aquatic organisms.

The community composition and toxigenicity of the diatom *Pseudo-nitzschia* in the open South Atlantic Ocean were characterized during the austral spring of 2007 by Guannel et al. (2015). Multiple morphological types of *Pseudo-nitzschia* were detected in coastal and in open-ocean waters. The toxin produced by *Pseudo-nitzschia*, DA, was present in at least 10 species in the South Atlantic (Gayoso, 2001; Reguera, 2002). Even though *Pseudo-nitzschia* sp. blooms occur in different environmental conditions, Marchetti et al. (2004) and Wells et al., 2005) showed a positive correlation between these blooming events and high levels of Fe(III) and ${\text{NO}}_{3}^{-}$, and low levels of ${\text{PO}}_{4}^{3-}$ and ${\text{SiO}}_{4}^{4-}$. Even more, differences in temperature, salinity, pH, high irradiance, and long-term photoperiod can affect the formation of *Pseudo-nitzschia* blooms (Lelong et al., 2012). Woods (2016) suggested a possible photo-oxidative stress regulation on DA's higher production under high irradiance. Cellular stress produced by these conditions may favor the production of toxins during HAB events. The main objective of this opinion article is to briefly report the toxicological implications of the harmful marine phycotoxin DA and its intrinsic properties. Special focus will be made on the reported oxidative stress status in marine algae in relation to the exposure to DA. Moreover, because Fe presence is known to be implicated in oxidative stress generation, and its occurrence seems to be a fundamental factor in the production of DA, the oxidative effects of the biotoxin will be discussed in relation to its capacity to bind Fe.

## Chemical, Biological, and Production Features of Da

DA is a tricarboxylic amino acid belonging to the category of cainoids (Wright and Quilliam, 1995). The main chemical features of DA (C_15_H_21_NO_6_) are as follows: average mass of 311.330 Da and percent composition of 57.87% C, 6.80% H, 4.50% N, and 30.83% O (Merck online index). Pure DA appears as colorless crystal needles. It is heat-stable and soluble in water, dilute mineral acids, and alkali solutions. It is slightly soluble in methanol and ethanol and insoluble in petroleum ether and benzene (Jenkins, 1996).

Even though DA was identified in 1975 as being produced from the Mediterranean macroalgae *Alsidium corallinum*, it was first isolated from the red alga *Chondria armata*. Its extracts have been used as an ascaricidal medication (Daigo, 1959) and as insecticide (Iverson and Truelove, 1994). DA was later found in either microalgae species (diatoms) or macroalgae species (red algae) (Ravn, 1995). This biotoxin was identified as a public health risk toxin after an incident occurred in 1987 on Prince Edward Island, Canada (Wright and Quilliam, 1995). It is recognized that mussels contaminated with high levels of DA from algae, when are consumed by humans, produce a severe disorder known as amnesic shellfish poisoning (ASP), which could even lead to the patient death (Pulido, 2008). However, no systematic information is known up to now about DA actions in photosynthetic organisms.

DA is an excitatory amino acid containing the structure of glutamic acid and resembles kainic acid (Todd, 1989). DA binds at the same receptor site in the central nervous system than kainic and glutamic acid (Mok et al., 2009), and its coexisting natural chemical analogs act as a potent excitatory neurotransmitter. Transmembrane absorption and biological barriers interaction with DA were reported. According to data provided by Preston and Hynie (1991), the blood–brain barrier greatly limits the amount of toxin that enters the brain in vertebrates. Kimura et al. (2011) studied the transcellular transport and intestinal absorption mechanism of DA through intestinal Caco-2 cellular monolayers. Their results suggested that the apical and basolateral transport of DA through these cells is mediated by anion transporters.

The biosynthesis pathway of the DA is not fully elucidated, but it is known that large amounts of ATP are required for its production (Pan et al., 1998; Thessen, 2007). Recently, Brunson et al. (2018) established a biosynthesis model by finding a cluster of genes related to recombinant DA biosynthetic enzymes and linked their mechanisms to the construction of a pyrrolidine skeleton. Moreover, Sobrinho et al. (2017) determined that DA concentrations of *P. multiseries* significantly increased under high Fe concentration, suggesting that Fe is required for the toxin synthesis. Extracellular DA in water undergoes photodegradation or biodegradation and does not accumulate in the water column (Bejarano et al., 2008; Zabaglo et al., 2016). However, the adsorption of AD in the sediment can have a long-term impact on the trophic web due to its transfer through benthic organisms (Burns and Ferry, 2007; Zabaglo et al., 2016).

## General Characteristics of Da on the Oxidative Metabolism

### Algal Ecology

Even though physiological and ecological roles for some marine toxins produced during HABs were postulated, the matter is not fully understood. Ding et al. (2007) suggested that responses of marine plants to adverse environmental conditions involve excess production of reactive O_2_ species (ROS). A cellular signaling, generated by the effects of the phycotoxins, results in a free radical cascade and activation of enzymatic processes. Consequently, an extensive damage of cell structures and ultimate cell death has been described (Aarts and Tymianski, 2004). Phytoplankton is responsible for the 50% of global primary production in the ocean, sustaining the pelagic food chains in the aquatic ecosystems (Roig, 2000), and for the substantial sink for CO_2_ in marine ecosystems. Then, if these organisms are adversely affected, the surrounding ecosystem may also feel the effects, either directly or indirectly, from the lack of a food source (Wang and Zheng, 2008).

### Hypotheses of DA Actions

Several hypotheses were presented to explain the potential roles for DA in the toxin productive algae: (1) Doucette et al. (2008) and Jackson et al. (1992) postulated that it could serve as an osmolyte under conditions of increasing salinity; (2) Tammilehto et al. (2015) suggested that it may act as a long sighted protection of the algae against the action of consumers such as copepods; (3) Trick et al. (2010) and Rue and Bruland (2001) proposed that DA could be a binding ligand for trace nutrients such as transition metals; (4) Xu et al. (2015) suggested that it may have allelopathic effects in other members of the phytoplankton community, stimulating changes in the dynamics and composition of this algae. Besides the possible combination of several of these actions, the DA effect on the rest of the photosynthetic community is an interesting point to investigate because its effect on non–toxin-productive aquatic organisms is not clear.

### New Methods of Inquiry

Allelopathic interactions between plants and other photosynthetic organisms showed both positive (hormesis) and negative (oxidative stress) effects, through the release of chemicals into the environment. These toxic effects include inhibition of growth of various organs and delay or restriction of seed germination (Abrahim et al., 2000). Oxidative stress could participate in the allelopathic response due to overproduction of ROS and alterations in the cellular antioxidant system (Abrahim et al., 2003; Bai et al., 2009). *Pseudo-nitzschia* cells can have allelopathic effects in sympathetic species (Granéli and Hansen, 2006). Lundholm et al. (2005) examined the potential allelopathic effect of pure DA additions to cultures of different phytoplankton species. In this study, the tested species were selected in order to represent different algal classes that occur in natural environments together with *P. multiseries*. The lack of allelopathic effects of the DA-producing marine diatom was reported under those experimental conditions. However, recent studies (Xu et al., 2015) have shown possible allelopathic effects in Fe-enrichment conditions in laboratory cultures (Prince et al., 2013; Sobrinho et al., 2017). Even more, Bates et al. (2018) suggested that DA could indirectly improve the competitive ability of *Pseudo-nitzschia* sp. on a phytoplankton community, and Fe is likely to be involved in this effect. Olson and Lessard (2010) argued that high concentrations of DA located within the diffusion zone of a cell can also affect microzooplankton grazing.

### Oxidative Effects Produced by DA

The generation of oxidative stress due to the exposure to DA has been reported in a large number of animal species, such as bivalves, fish, and aquatic and terrestrial mammals, as a secondary effect of its neurotoxicity (Zabaglo et al., 2016). Increased ROS formation was also observed in the nematode *Caenorhabditis elegans* when it was exposed to DA (Tian and Zhang, 2019). Moreover, an antioxidant treatment suppressed the toxic effects of the DA on the locomotion behavior of the nematodes, suggesting that oxidative stress is possibly involved in the mechanism of DA toxicity. In photosynthetic organism, the oxidative condition of the pennate diatom *Phaeodactilum tricornutum*, a non–toxin-producing microalgae, was characterized during the exposure to DA under laboratory conditions. The reported evidence suggested that when *P. tricornutum* was exposed to DA, (a) the reactive species generation rate was increased in the intracellular environment (Cabrera et al., 2019a), and (b) the reactive species were released to the extracellular medium (Cabrera et al., 2019b,c). Cabrera et al. (2019a) reported DA effects in terms of oxidative status of *P. tricornutum* during the exponential (EXP) phase, incubated in the presence of 64 μM DA. The generation of active species was measured following the oxidation rate of 2′,7′-dichlorofluorescein diacetate (DCFH-DA). The linear increase in the production of reactive species from both control and exposed algae homogenates was significantly higher in cells incubated in the presence of DA, as compared to control ones. Also, the reaction rate was measured in the presence of scavengers, suggesting a similar contribution of several active species (H_2_O_2_, Fe, and ${\text{O}}_{2}^{-}$). To study the effects on the extracellular environment, *P. tricornutum* cells were exposed to DA, washed with buffer, and incubated with DCFH-DA. After isolation of the cells by centrifugation, the release of reactive species to the supernatant was assessed by the oxidation rate of DCFH-DA using either control or cells previously exposed to DA. An increase by three-fold was reported in treated cells in the EXP phase of development, as compared to control ones. This oxidation rate was decreased in the presence of superoxide dismutase, catalase, dimethyl sulfoxide, desferroxamine, and glutathione (Cabrera et al., 2019b,c). Similar profiles were observed in either LAG or stationary (ST) phases of growth of the cells previously incubated with DA. Moreover, many studies reported dissolved DA in seawater (de la Iglesia et al., 2008; Mafra et al., 2009; Vera-Avila et al., 2011; Wang et al., 2012; Godinho et al., 2018) showed that during blooms of *P. multiseries* dissolved DA could be detected in the culture medium. These results could suggest that waterborne exposure of marine organism should be considered in a macroscale situation such as algal blooms. Taking into consideration this possible scenario, the enhanced production of DA during HABs could increase the release of DA to the aquatic environment, and the effects on oxidative stress conditions on other members of the community could be affected.

## Discussion

**Figure 1:**
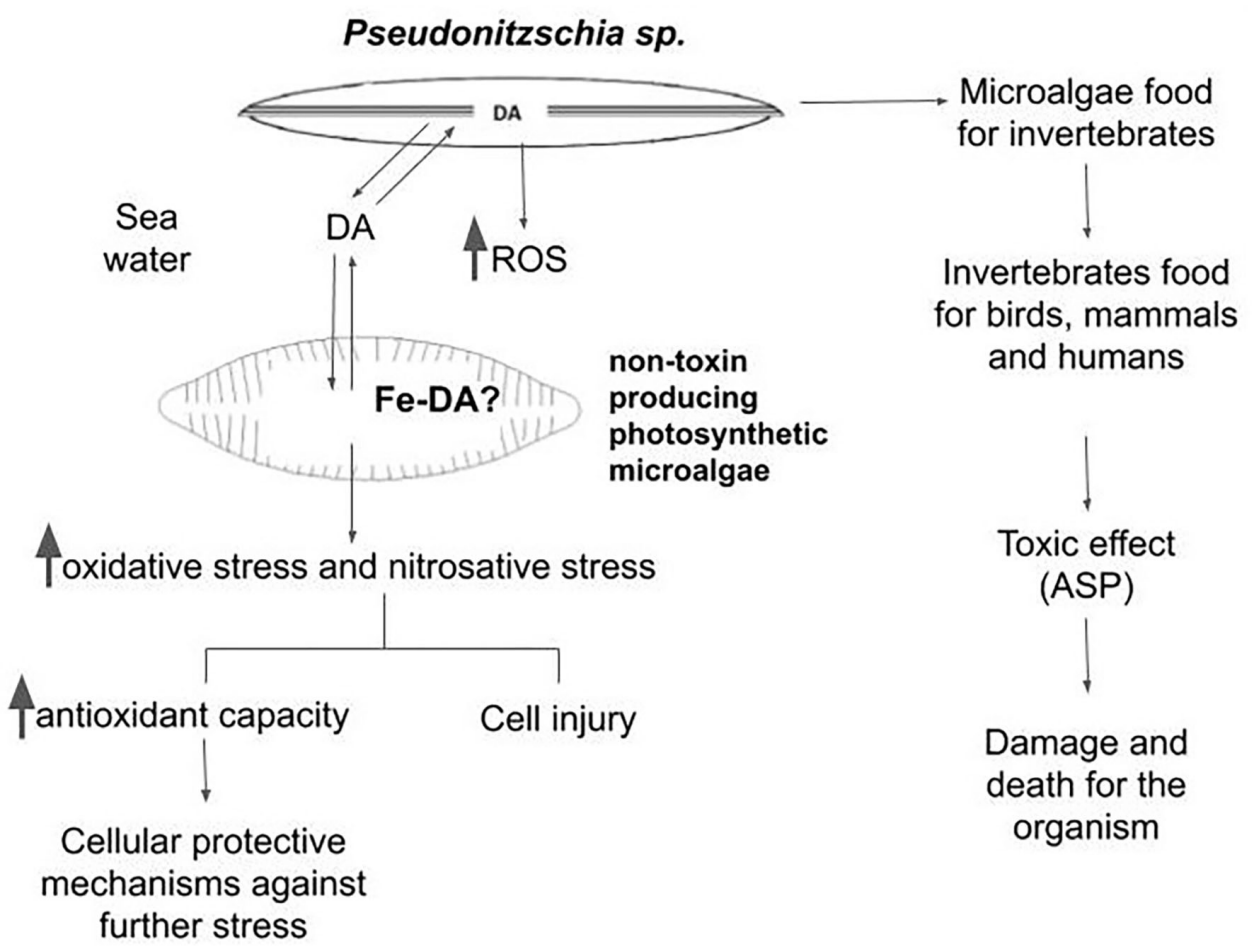
Schematic diagram showing the possible interactions between the phycotoxin-producing microalgae and the web community in aquatic environments.

Alterations of the nutrient influx in marine water due to climate change could modify HAB frequency and magnitude, increasing its impacts. The diagram shown in Figure 1 briefly describes a possible scenario in the aquatic environment. On the one hand, when *Pseudo-nitzschia* cells produce DA in considerable amounts, the toxin gets into the food chain. When this occurs, DA could lead to the death of certain consumers. In humans, the poisoning was characterized by a constellation of clinical symptoms and signs, involving multiple organ systems, including the gastrointestinal tract, the central nervous system, and the cardiovascular system. Among the most prominent features described was memory impairment, which led to ASP, which could be even lethal.

On the other hand, DA production starts in late EXP and ST phase of the *Pseudo-nitzschia* growth cycle (Lelong et al., 2012). In the late ST phase, cells are physiologically impaired by injury in the structure of the wall and in the membrane (Pan et al., 1996). Therefore, DA tends to be released into the culture medium, along with ROS generated by the cells (as shown by Cabrera et al., 2019b,c). Because of the short life span of ROS, this increased production could not be very effective in damaging other organisms in the aquatic community. However, the release of DA could allopathically affect (protect or injure) other species of non–toxin-producing photosynthetic microalgae. Using a highly sensitive adsorptive cathodic stripping voltammetric technique, Rue and Bruland (2001) showed that DA forms Fe chelates with a conditional stability constant of K_condFeDA,Fe(III)_ = 10^8.7±0.5^ M^−1^. The degree to which the DA acts as a trace metal chelator reveals that it has the potential to modify the availability of these metals in the seawater. Thus, the physiological role of DA for toxigenic *Pseudo-nitzschia* species may be tied closely to the acquisition of Fe in coastal waters. Among several other postulations, the formation of a Fe–DA complex could be a very attractive hypothesis to explain the increased generation of ROS both in producing and non–phycotoxin-generating species. Chemical aspects of the Fe chelators, such as Fe affinity, Fe selectivity, molecular weight, and lipophilicity, in addition to stability and redox properties of the resultant Fe complex, drastically change the ability of the Fe complex to catalyze radical generation. The presence of a catalytically active Fe–DA complex could favor lipid peroxidation and radical generation in a significative amount, by affecting the composition of the labile Fe content within the cells. This is a novel point of view that should be further explored. The capacity of the antioxidant defense in each species will be the main factor to determine the hormetic (beneficial) or the damaging effect of the triggering of the burst of oxidative species.

However, other alternatives could be considered under changing environmental conditions that can modify DA-dependent active species generation in aquatic organisms. Hypoxia is a common condition in aquatic environments, and exposure to hypoxia followed by reoxygenation is often believed to induce oxidative stress and activation of relevant signaling molecules such as the hypoxia-inducible factor 1α. This transcription factor is a potent coordinator of acclimation processes in various stress conditions. Eutrophication and extensive algal blooms caused by anthropogenic activity (input of nutrients, fertilizers, and human waste) deplete the ocean and lake bottoms of O_2_, i.e., make them hypoxic. In the seas, the large hypoxic areas are also known as hypoxic dead zones, and they are expanding quickly (Conley et al., 2009). Hypoxic bottoms release phosphorus from the aquatic sediments, which feed the algae even further. Hypoxia has an acute effect on the benthic fauna, and if the hypoxic environments are allowed to expand further, the whole ecosystem will be disturbed (Conley et al., 2009; Conley, 2012). Recently, Borowiec and Scott (2020) reported that hypoxia acclimation of killifish leads to adjustments in ROS homeostasis and oxidative status that do not reflect oxidative stress, but may instead be part of the suite of responses used to cope with chronic hypoxia. Thus, *in situ* studies could show the alterations due to other transitory changes in the environment that may occur during the HABs. Overall, the specific cellular response to the increased oxidative stress triggered by DA will be one of the important factors to allow survival of each organism that contribute to determine how the composition of the community will be affected by an increasing magnitude of the HAB season produced by the climate global change.

## Author Contributions

JC contributed to the bibliographic search and in the manuscript writing. PG contributed to the manuscript writing. SP participated in the designing of the original opinion idea and in the manuscript writing. All authors contributed to the article and approved the submitted version.

## Conflict of Interest

The authors declare that the research was conducted in the absence of any commercial or financial relationships that could be construed as a potential conflict of interest.
